# Quantitative comparison of protein-protein interaction interface using physicochemical feature-based descriptors of surface patches

**DOI:** 10.3389/fmolb.2023.1110567

**Published:** 2023-02-06

**Authors:** Woong-Hee Shin, Keiko Kumazawa, Kenichiro Imai, Takatsugu Hirokawa, Daisuke Kihara

**Affiliations:** ^1^ Department of Chemistry Education, Sunchon National University, Suncheon, South Korea; ^2^ Department of Advanced Components and Materials Engineering, Sunchon National University, Suncheon, South Korea; ^3^ Pharmaceutical Discovery Research Laboratories, Teijin Pharma Limited, Tokyo, Japan; ^4^ Cellular and Molecular Biotechnology Research Institute, National Institute of Advanced Industrial Science and Technology, Tokyo, Japan; ^5^ Division of Biomedical Science, Faculty of Medicine, University of Tsukuba, Tsukuba, Japan; ^6^ Transborder Medical Research Center, University of Tsukuba, Tsukuba, Japan; ^7^ Department of Biological Sciences, Purdue University, West Lafayette, IN, United States; ^8^ Department of Computer Science, Purdue University, West Lafayette, IN, United States; ^9^ Center for Cancer Research, Purdue University, West Lafayette, IN, United States

**Keywords:** protein-protein interaction, PPI, PPI drugs, molecular surface, protein-protein interaction (PPI), 3D Zernike descriptor

## Abstract

Driving mechanisms of many biological functions in a cell include physical interactions of proteins. As protein-protein interactions (PPIs) are also important in disease development, protein-protein interactions are highlighted in the pharmaceutical industry as possible therapeutic targets in recent years. To understand the variety of protein-protein interactions in a proteome, it is essential to establish a method that can identify similarity and dissimilarity between protein-protein interactions for inferring the binding of similar molecules, including drugs and other proteins. In this study, we developed a novel method, protein-protein interaction-Surfer, which compares and quantifies similarity of local surface regions of protein-protein interactions. protein-protein interaction-Surfer represents a protein-protein interaction surface with overlapping surface patches, each of which is described with a three-dimensional Zernike descriptor (3DZD), a compact mathematical representation of 3D function. 3DZD captures both the 3D shape and physicochemical properties of the protein surface. The performance of protein-protein interaction-Surfer was benchmarked on datasets of protein-protein interactions, where we were able to show that protein-protein interaction-Surfer finds similar potential drug binding regions that do not share sequence and structure similarity. protein-protein interaction-Surfer is available at https://kiharalab.org/ppi-surfer.

## 1 Introduction

Proteins are involved in almost all essential biological processes. Biological functions of proteins are usually exhibited through interaction with other molecules such as DNA, proteins, hormones, and small chemical compounds. Therefore, a core concept of drug discovery is to control the interaction of proteins with other biomolecules. Traditionally, a small chemical compound was identified as a drug that binds to a well-defined pocket of a target protein. The compound typically competes with a natural binder of the target to inhibit or enhance its function. Even though the concept has been successfully applied to drug discovery since the mid-twentieth century, the efficiency of the research and development process has been decreasing (Scannell et al., 2012). The main reason is because the space of druggable proteins explored has almost been saturated (Rask-Andersen et al., 2011; Scannell et al., 2012). According to Rask-Andersen et al. (2011), the number of FDA-approved drugs targeting unexploited proteins has decreased from ten to five from 2001 to 2010. To expand druggable sites in the human proteome, protein-protein interactions (PPIs) have been suggested as a new type of drug targets since early 2000 (Toogood, 2002; Arkin and Wells, 2004; Ivanov et al., 2013; Jin et al., 2014). Since an interaction is generated with more than two proteins, the number of PPIs is much greater than the single protein drug target space. The size of the human proteome is estimated at about 19,000, whereas the size of the PPI interactome is approximated as 650,000 by Stumpf et al. (2008).

A typical example of small molecule protein-protein interaction inhibitors (SMPPIIs) is those which target interaction between p53 and mouse double mutant 2 homolog (MDM2). P53 is a tumor suppressor but it is downregulated in cancer cells *via* interaction with MDM2 (Vassilev et al., 2004). Thus, compounds that bind at the PPI site of MDM2 can prevent MDM2 to interact with p53 and re-activate p53. Many compounds were developed under this strategy. In fact, over 300 small chemical compounds with an IC50 value less than 1 nM are reported in the ChEMBL compound database (Gaulton et al., 2017).

Although PPIs have attracted lots of attention as drug targets, there are not many SMPPIIs successfully developed so far. From 2004 to 2014, about forty PPIs have been targeted, among which only six of them have further proceeded to clinical trials (Shin et al., 2020). PPIs are still difficult to target because they have different nature from traditional drug-binding sites. Drug-binding interface at PPI tends to be larger, flatter, and more hydrophobic than single protein targets. Moreover, a drug binding site at PPI is often formed by transient surface fluctuation, which is not observed in the protein-protein complex. Due to these differences, PPI is more challenging for discovering pharmacological compounds (Arkin et al., 2003; Eyrisch and Helms, 2007). Alzyoud et al. (2022) calculated the druggability scores (Dscore) of twelve commonly targeted PPIs using SiteMap. Out of six PPIs where both apo structure and SMPPII bound conformation are available, the Dscore of the apo structure turned out to be smaller than the corresponding holo form. One interesting example is B-cell lymphoma-extra large. The Dscores of the holo and apo structures are very different, 1.09 and 0.73, respectively. Induced fit occurs on F105, L108, and L130, which causes unwinding of a helix and formation of a groove near the compound binding pocket, which is not observed in the apo structure.

SMPPIIs also have distinguishing features from traditional drugs, which are summarized as the rule of four (RO4): SMPPIIs tend to have a molecular weight higher than 400 Da, logP higher than four, more than four rings, and more than four hydrogen-bond acceptors (Morelli et al., 2011). These properties are very different from the well-known Lipinski’s rule of 5 (Lipinski, 2004) for traditional drugs that bind to a pocket in a protein surface. As PPIs have different properties than traditional druggable sites, computational tools are urgently needed that can characterize, compare, and classify PPI sites so that researchers can identify potential druggable PPI sites and repurpose SMPPIIs. However, computational methods for developing SMPPIIs are critically lacking (Shin et al., 2017).

Several computational algorithms have been developed to examine and classify PPIs. The methods can be categorized into two classes, ones that align PPIs first and the others which are alignment-free. One of the alignment-based methods is MAPPIS (Shulman-Peleg et al., 2007). The method aligns PPIs and identifies corresponding amino acids that have common interaction types from the PPIs, such as hydrogen bonds, hydrophobic, and aromatic interactions. PCalign (Cheng et al., 2015) quantifies physico-chemical similarities between amino acids at PPIs. The program gives a score called PC-score that quantifies the similarity of PPIs based on the classification. Gao and Skolnick developed an alignment-based PPI comparison algorithm, iAlign (Gao and Skolnick, 2010). The Galinter method (Zhu et al., 2008) represents a PPI as a graph that connects non-covalent interactions as edges and aligns two PPIs with a graph matching algorithm.

PatchBag is an example of alignment-free methods (Budowski-Tal et al., 2018). In PatchBag, an exposed residue is represented as a normal vector of a local surface patch that is defined as neighboring residues. Patches are classified by geometrical similarity of residues in patches, which are then used to define similarity of PPIs that are represented by a set of patches. PBSword (Pang et al., 2012) is another alignment-free method, which represents a PPI as a set of vectors of local geometric features.

Recently, we have developed a series of surface-based molecule similarity calculation programs using three-dimensional Zernike descriptors (3DZD) (Venkatraman et al., 2009a; Kihara et al., 2011). The notable strength of 3DZD is that it transforms a molecular surface into a rotationally-invariant vector with the Canterakis-Zernike base function. 3DZD allows fast comparison of molecular surfaces in terms of their shapes and physicochemical properties because similarity is computed by comparing vectors. Taking advantage of the strength, 3DZD has been applied for many biological problems including protein shape comparison (La et al., 2009; Xiong et al., 2014; Han et al., 2017; Aderinwale et al., 2022), protein-protein docking (Christoffer et al., 2021a; Christoffer et al., 2021b), comparing chemical compounds (Venkatraman et al., 2009b; Shin et al., 2015), structure-based virtual screening (Hu et al., 2014; Shin et al., 2016; Shin and Kihara, 2018), cryo-EM map comparison (Sael and Kihara, 2010a; Esquivel-RodriguezXiong et al., 2015; Han et al., 2017; Han et al., 2019), and binding site comparison (Sael and Kihara, 2010b; Chikhi et al., 2011; Sael and Kihara, 2012; Zhu et al., 2015; Zhu et al., 2016). Particularly, for aiding drug discovery, we have developed a virtual screening program, PL-PatchSurfer (Hu et al., 2014; Shin et al., 2016; Chiba et al., 2017; Shin and Kihara, 2018; Chiba et al., 2019; Shin and Kihara, 2019). PL-PatchSurfer calculates complementarity between a protein binding pocket and a ligand compound using 3DZD. In PL-PatchSurfer molecular surface of a binding pocket and a ligand is segmented into a set of overlapping surface patches, where local chemical complementarity is represented by 3DZDs. The fit of a binding pocket and a ligand is evaluated by finding patch pairs from the pocket and the ligand that are complementary to each other. PL-PatchSurfer showed superior performance to existing methods in virtual screening especially when the input receptor structure is slightly different from the holo form, such as an apo structure or a computational model because the surface representation is more tolerant to such structural changes.

Here, we developed a new method, PPI-Surfer, which represents a PPI surface with 3DZDs and quantifies the similarity between different PPIs. Similar to PL-PatchSurfer, PPIs are segmented into overlapping surface patches and their physicochemical properties are represented by 3DZDs. Compared with the above-mentioned PPI comparison methods, PPI-Surfer is unique in that several physicochemical features mapped on a PPI surface are naturally described in the same fashion by using 3DZDs. Compared with existing methods discussed above, first, PPI-Surfer is an alignment-free method, thus different from the alignment-based methods which need to superimpose interacting protein complexes to compute their similarity. Compared with the two existing alignment-free methods (Pang et al., 2012; Budowski-Tal et al., 2018), a novel strength of PPI-Surfer is it can identify local surface similarities in PPIs because combinations of similar surface patches within the two given PPIs are explored. In contrast, the two existing methods compute similarity of a pre-defined PPI regions.

PPI-Surfer was benchmarked on three datasets: On the 2P2I database (Morelli et al., 2011; Basse et al., 2016), which stores 32 experimentally determined complex structures of drug-targeted PPIs, PPI-Surfer overall identifies similarities of PPIs consistently as a sequence-based and a ligand docking-based method yet was able to identify PPI site pairs that are similar in terms of the surface properties but not similar in terms of sequence. Then, we applied PPI-Surfer to identify hotspot regions of PPIs that are identified by MAPPIS (Shulman-Peleg et al., 2007), an alignment-based PPI comparison method. Finally, we used a dataset of SARS-CoV-2 spike protein binders (Cao et al., 2020), and showed the program successfully identified the true binder from artificially generated decoy proteins.

## 2 Materials and methods

### 2.1 The algorithm of PPI-Surfer

#### 2.1.1 Generating of molecular surface patches

**FIGURE 1:**
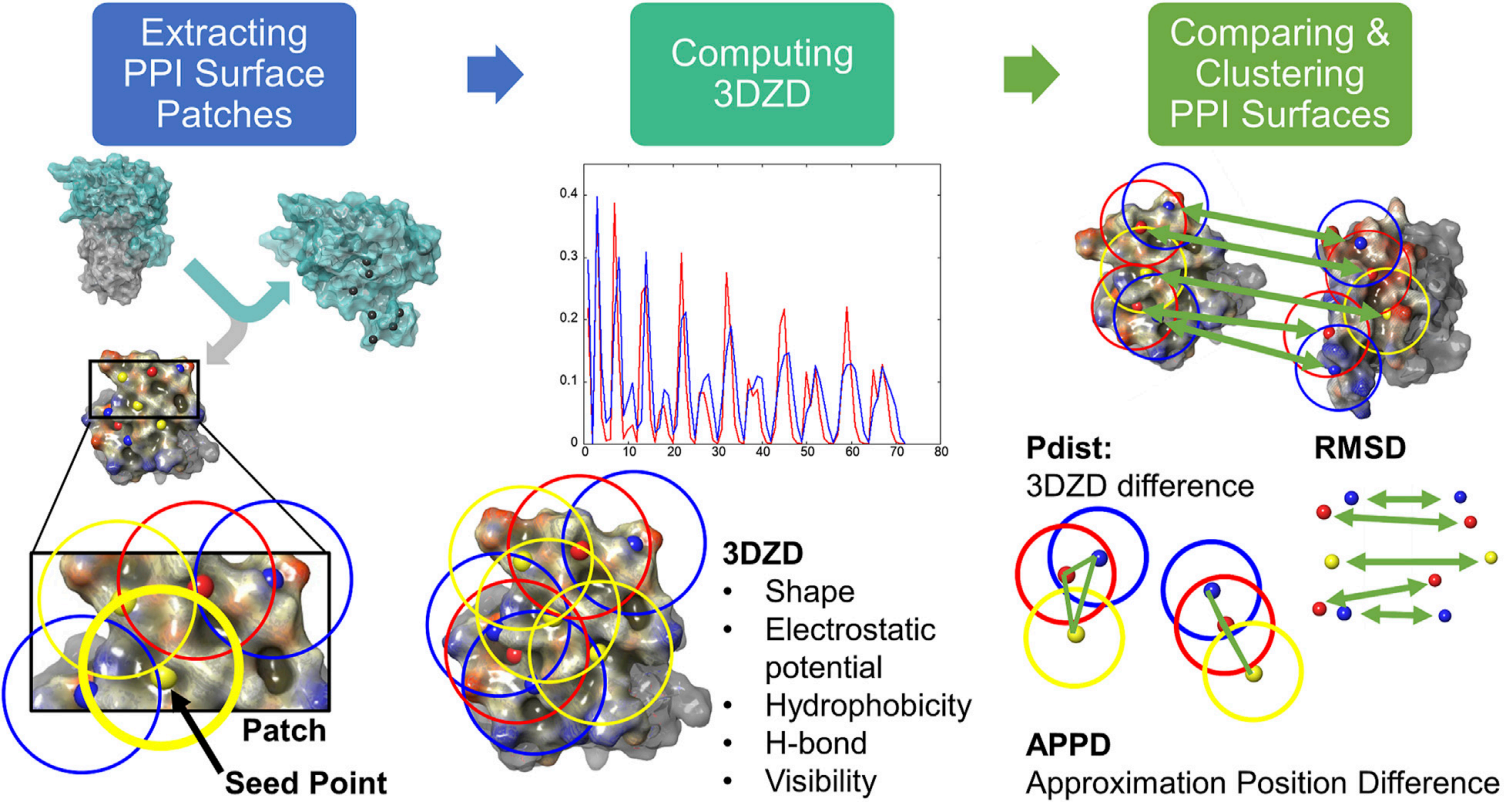
Workflow of PPI-Surfer.

The aim of the PPI-Surfer is to calculate the similarity between two PPIs. A PPI surface is defined by surface atoms that are closer than 5 Å from any heavy atoms of the binding partner protein. Figure 1 illustrates an overview of the algorithm. The first step of PPI-Surfer is to generate surface patches of given PPI structures using the APBS program (Jurrus et al., 2018). APBS constructs a 3D grid spacing of 0.6 Å, covering the whole structure of a given protein. The program constructs the surface of a protein by removing the grid points outside of the protein with a water probe (a 1.4 Å radius). For instance, for the structure of SRC kinase, which has 452 amino acids, the number of grid points is reduced to 300,044 from about six million points. Then, the electrostatic potential on surface points is assigned by solving the Poisson-Boltzmann equation. To further characterize protein surface, we also compute and assign three other physicochemical features, hydrogen-bond acceptors and donors, hydrophobicity, and visibility. For hydrophobicity, atomic logP values are assigned to each atom using the same parameter as the XlogP3 program (Cheng et al., 2007). Then, a molecular hydrophobic field at a surface point is calculated as Eq. 1 (Heiden et al., 1993).

$${MHP}_{i}=\frac{\sum_{j=1}^{N}f_{j}{\left[1+\exp\left(r_{ij}-4\right)\right]}^{-1}}{\sum_{j=1}^{N}{\left[1+\exp\left(r_{ij}-4\right)\right]}^{-1}}$$ (1)

Indices *i* and *j* are a surface point and an atom from a protein, respectively. *f*_*j*_ is an atomic logP value from the XlogP3 parameter and *r*_*ij*_ is the distance between the voxel point *i* and the atom *j*.

The hydrogen-bond property of a surface point is assigned by considering the closest atom of the protein. If the closest atom is a hydrogen-bond donor or an acceptor, a value of 1 or −1, respectively, is assigned to the surface point. Otherwise, the point is assigned with zero.

The visibility concerns the local curvature of a surface point (Li et al., 2008; Sael and Kihara, 2012). It has a value that ranges from 0 to 1 with 1 for a fully exposed and 0 for a fully buried point. To compute visibility for a surface point, a set of rays is expanded from the point to 512 directions and the fraction of rays that are not blocked by a protein surface is computed. A ray is considered blocked if the nearest-neighbor point (which is in 0.3 Å) belongs to the protein surface.

After assigning all the features to surface points the surface of a given PPI surface is divided into patches. Seed points are iteratively selected from protein surface points so that they are separated by more than 5 Å from each other. Then, a surface patch is segmented by a sphere of a 10 Å radius centered at a seed point. Patches can overlap and cover the entire PPI surface (see Figure 1).

The physicochemical properties on a surface patch are represented by 3DZD (Canterakis, 1999; Venkatraman et al., 2009a; Kihara et al., 2011) essentially in the same way as we used in PL-PatchSurfer2 (Shin et al., 2016; Shin and Kihara, 2018). 3DZD represents a 3D function f(**x**) as a vector of coefficients. The similarity (or the distance) of two 3D functions, which represents a physicochemical feature on patch surfaces, can be simply computed by the Euclidean distance between the vectors. A 3D function f(**x**) is converted to 3DZD as follows:

$$\Omega_{nl}^{m}=\frac{3}{4\pi }\int_{\left|x\right|\leq 1}f\left(x\right)Z_{nl}^{m}\left(x\right)dx$$ (2)

$x=\left(x,y,z\right)=\left(r,\theta ,\pi \right)$ and Z(**x**) is the Zernike-Canterakis basis function, composed of radial function $R_{nl}\left(r\right)$ and spherical harmonics $Y_{l}^{m}\left(\theta ,\varphi \right)$ (Eq. 3).

$$Z_{nl}^{m}=R_{nl}\left(r\right)Y_{l}^{m}\left(\theta ,\varphi \right)$$ (3)

*n*, *l*, *m* are integers and the conditions for the numbers are −l < *m* < *l*, 0 < *l* < *n*, and (*n-l*) is even.

3D Zernike moment is further converted to 3DZD, which is rotationally invariant, by taking a norm:

$$F_{nl}=\sqrt{\overset{m=l}{\underset{m=-l}{\sum }}{\left(\Omega_{nl}^{m}\right)}^{2}}$$ (4)

The dimension of 3DZD is determined by the order *n*, which also sets the resolution of the 3DZD representation. In PPI-Surfer, *n* is set to 15, making 3DZD a 72-dimensional vector.

#### 2.1.2 Similarity between PPI sites

To quantify similarity of two PPI sites, which are represented by a set of patches, we optimize pairing of patches from the two PPI sites so that the following score (distance), PatchScore, is minimized:

$$Patch\;Score\left({PPI}_{1},{PPI}_{2}\right)=w_{P}\times pDist\left({PPI}_{1},{PPI}_{2}\right)+w_{R}\times RMSD\left({PPI}_{1},{PPI}_{2}\right)+w_{A}\times APPD\left({PPI}_{1},{PPI}_{2}\right)$$ (5)

The first term, *pDist*, is a weighted sum of the Euclidean distance between 3DZD for matched patch A and B.

$$pDist\left({PPI}_{1},{PPI}_{2}\right)=\underset{i}{\sum }w_{i}\left|{3DZD}_{1,i}-{3DZD}_{2,i}\right|$$ (6)

The index *i* denotes physicochemical features of a patch, which are 3D shape, electrostatic potential, visibility, hydrogen-bond acceptors/donor distribution, and hydrophobicity. Relative weights of the features, *w*_*i*_, were trained on the protein structures that bind to multiple partner proteins, called a hub protein set, extracted from the PiSite database (Higurashi et al., 2009). PiSite collects structures of protein complexes that share a common component protein and provides protein-protein interaction sites at a residue level. The details of the training will be described in the next section.

The second term of Eq. 5 is the root-mean-square deviation (RMSD) of the seed points of the matched patches. The coordinates of seed points of matched patches on each PPI site are extracted and superimposed to calculate the RMSD. The last term of Eq. 5 is called *APPD*, an abbreviation of Approximate Patch Position Difference:

$$APPD\left({PPI}_{1},{PPI}_{2}\right)=\left|{APP}_{1}-{APP}_{2}\right|$$ (7)

*APP* is a histogram of the geodesic distance from a seed point to other seed points in the given PPI site. The bin size was set to 1.0 Å. *APP* represents an approximate position of a patch in the PPI surface, i.e., the patch is placed in the middle or edge of a PPI site.

To search similar patch pairs between PPI sites, a modified version of the auction algorithm, a bipartite matching method, is used (Sael and Kihara, 2010b). The algorithm minimizes the PatchScore (Eq. 5) by matching similar patches pairs iteratively. Once the correspondence of surface patches is finalized, the overall similarity between the two PPI sites is calculated as a different score, the PPI Score:

$$PPI\;Score=k_{P}\times Avg\left(pDist\right)+k_{R}\times Avg\left(RMSD\right)+k_{A}\times Avg\left(APPD\right)+k_{S}\times SD$$ (8)

where the *Avg(X)* is an average of a term X in Eq. 5 over all matched pairs. All the weights (*k*_*i*_ *s*) in Eq. 8 were optimized to maximize the benchmark performance measured by the hub protein set, which will be described in the next section. The last term, *SD*, refers to the Size Difference between the PPI sites, which is inferred by counting the number of patches on each PPI site. The term is determined by dividing the difference between the number of patches of two PPI sites by the number of surface patches of the larger PPI site. PPI Score is a distance metric; similar PPI sites have a small value.

### 2.2 Score optimization using the hub protein dataset

The weight optimization of the scores was performed on the hub protein dataset. This dataset contains “hub” proteins, which bind multiple partner proteins using the same interface. We first obtained 180 complexes that consist of 69 hub proteins and their binding partners from PiSite (Higurashi et al., 2009). From this set, the complex with the highest resolution was selected and the rest were disregarded if multiple structures of the same complex were included. Also, we removed proteins that undergo a large conformational change upon binding. An example of this category of the hub protein is calmodulin. Finally, a hub protein and its binding proteins were removed if the binding proteins do not share a sufficiently large common binding area with the hub protein. To quantify the common PPI area in the two binding proteins, first, two complex structures between the hub and a binding protein were overlapped by superimposing the hub protein structures. Then, the PPI interfaces and surface patches in the two binding proteins were generated. Patches from the two binding proteins were considered as corresponding overlapping patches if the distance between their seed points on the PPI surfaces was less than 5 Å. If the number of overlapping patch pairs was less than five, the partners were discarded. A hub protein was kept in the training set if it has more than two partner proteins. After these steps the dataset remained 129 PPI pairs of 49 hub proteins and their interacting proteins. The number of interacting proteins for a hub protein ranged from 2 to 6 (average: 2.6).

Using the hub protein dataset, weights in Eqs 5, 6, 8 were optimized. First, the weight values for *pDist* (Eq. 6) were optimized, which concerns the balance among given features that characterize surface patches. We aimed to identify weights that can find corresponding PPI patches in the binding proteins that share the same hub proteins. The corresponding PPI patches were defined as the distance between seed points is less than 5 Å.

The *pDist* weight optimization was performed as follows: First, we counted the total number of corresponding PPI patches after superimposing two PPI surfaces with hub protein. Then, the weight sum of Eq. 6 was computed for all pairs of patches from the two PPI sites. From a query patch of a PPI site, we examined whether the patch from the other PPI sites with the minimum distance was its corresponding patch or not. All weights were trained by a grid search, by varying the value from 0.0 to 2.0 with an interval of 0.1. The target function of the training is the number of identified corresponding pairs by *pDist* divided by the total number of corresponding pairs that are found at the first step. The resulting weights were 1.0, 0.1, 1.0, 0.3, and 1.6 for shape, the electrostatic potential, visibility, hydrogen-bond acceptor/donor, and hydrophobicity, respectively. We took the grid search approach rather than a machine learning approach to determine the weights because the number of features and the number of training data were not large.

Next, the weights of Eq. 5 were trained. The hub protein set and the grid search strategy were also used for this step. The weights of individual terms searched from 0.0 to 2.0 with a grid space of 0.1. Auction algorithm was applied to identify patch pairs that have minimum Patch Score (Eq. 5). The target function was the number of the corresponding patches identified by Auction algorithm divided by the total number of corresponding patches. The success rate of 19.99%. The optimized weights are 0.7, 0.9, and 1.7 for *pDist*, RMSD, and *APPD*, respectively.

Lastly, weights for the PPI Score (Eq. 8) were determined. The weights were optimized so that a query PPI surface (the PPI surface of a binding protein) was able to identify the similar PPI sites, which is the PPI site of a binding protein that shares the same hub protein among all the 158 binding proteins in the hub dataset. The target function we used was an average rank of the correct partner proteins that share the same hub protein. For example, if the hub protein H interacts with partner proteins A, B, C, D, and E, we took A as a query and searched the dataset that includes the other partner proteins. Then, the average rank of the other four binding proteins, B, C, D, and E, was calculated. For this test we only used 20 hub proteins that have more than three partner proteins were used. The best average rank is 44.036. The optimized weights of individual terms of Eq. 8 are 0.6, 0.1, 0.2, and 0.1 for *pDist*, RMSD, *APPD*, and *SD*, respectively. PPI-Surfer is made available at https://kiharalab.org/ppi-surfer.

## 3 Result and discussion

The developed PPI-Surfer was tested in classifying PPI surfaces in three datasets. The three datasets have no overlap with the hub protein set taken from PiSite that were used as the training set to determine weighting factors in the scoring functions of PPI-Surfer.

### 3.1 Application of PPI-Surfer to classify PPI surfaces in the 2P2I database

**FIGURE 2:**
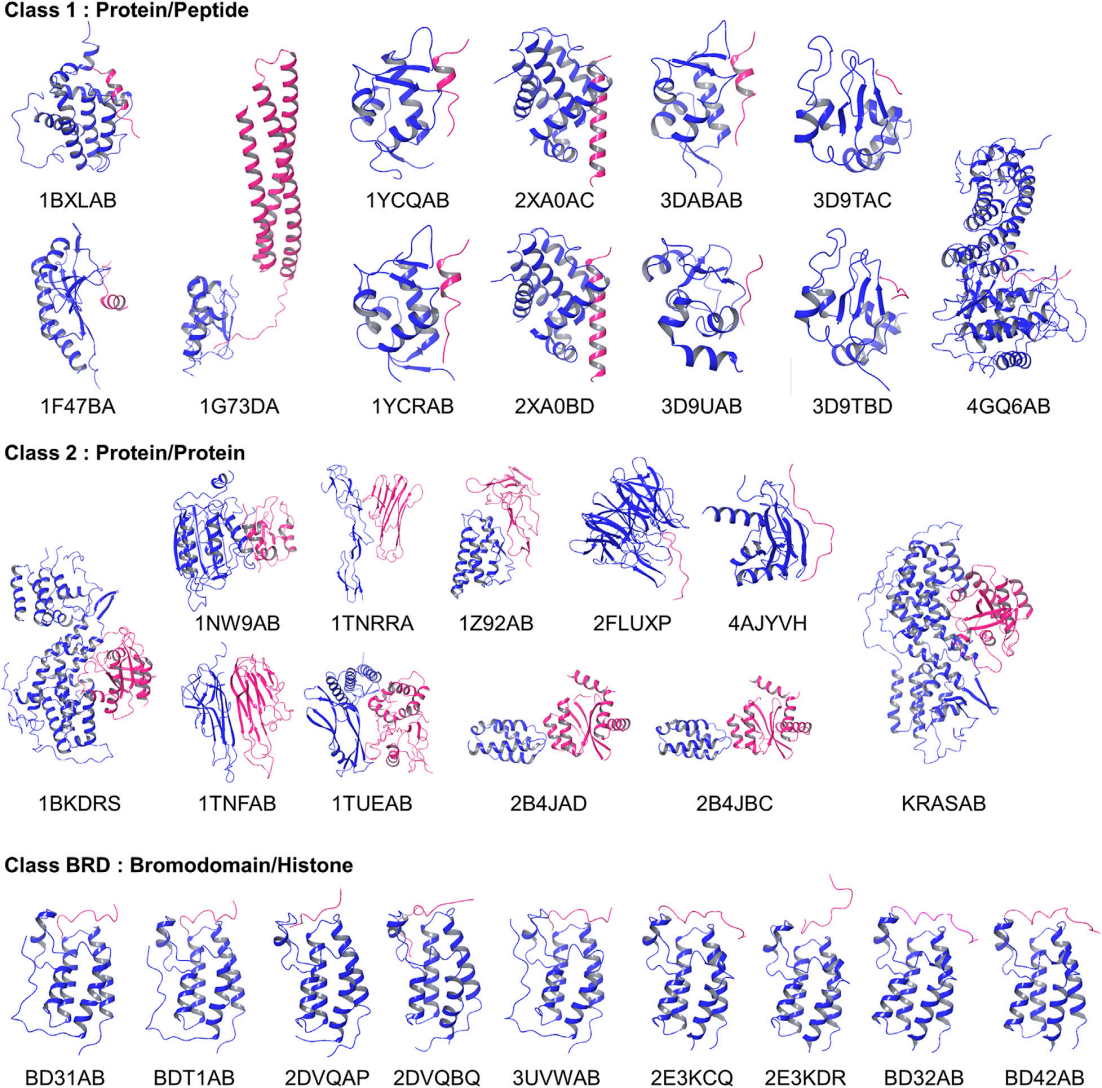
Entries in the 2P2I dataset. Proteins in blue were considered as receptors and their PPI surface were compared.

The 2P2I database is a hand-curated database of 3D structures of druggable PPI interfaces (Morelli et al., 2011; Basse et al., 2016). It contains 32 PPIs (Accessed on September 2017). For each PPI, three types of structures are provided, which are a PPI complex, protein-ligand (a drug molecule) complexes, and apo protein structures of a receptor protein that the drug and partner protein bind. The database classifies PPI surfaces into three categories: protein-peptide complexes, protein-protein complexes, and bromodomain-histone complexes. The last category, bromodomain-histone, could be included in the first category, protein-peptide complexes. However, the authors classified them into a separate class because they share specific binding modes and high sequence similarities. In addition, compounds that inhibit the bromodomains have a smaller size and lower hydrophobicity than inhibitors of the other categories. We analyzed the interface of the receptors taken in each of the 32 PPIs, which is the one that has protein-bound structures. The 32 complex structures are shown in Figure 2.

To understand how PPI Score evaluates the similarity of PPI sites (Eq. 8), we computed the PPI Score for all pairs of the 32 PPI sites using PPI-Surfer and compared the results with two other reference scores that quantify different aspects of PPIs. The first reference we used was the sequence similarity of the target proteins, which is a conventional metric to capture evolutional and the global similarity of proteins. We used the bit scores of the SSEARCH program (Pearson, 1991) for sequence similarity. Then, for a single PPI site, a vector of bit scores against all PPI sites in the dataset was constructed. The sequence distance between PPI sites was defined as 1.0—(cosine of the vectors of the two PPI sites). Thus, if two PPI sites are identical, the sequence distance is 0.0.

The second reference score we compared against with the PPI Score considers the similarity of the ranked order of drugs that would bind to PPI sites. This score was used in previous works for comparing drug-binding pockets by other groups (Fukunishi et al., 2005; Govindaraj and Brylinski, 2018). We call it the drug ranking distance. The assumption of this score is that similar protein surface would bind similar ligands. Thus, the drug ranking distance evaluates PPI sites focusing on a different aspect from the PPI score by PPI-Surfer. To compute the drug ranking distance, we docked 1,267 FDA-approved drugs from the Prestwick Chemical Library (https://www.prestwickchemical.com/), a collection of 1,520 off-patent small molecules, to the PPI sites using the GLIDE SP ligand docking program (Halgren et al., 2004). Among the 1,267 drugs, 1,113 were successfully docked to all the 32 PPIs. To run GLIDE SP the inner and outer docking boxes were set to have 10 Å and 30 Å lengths for each side and the centers of the docking boxes were set to the centroid of the PPI interface residues. The interface residue was defined as residues that are closer than 4.5 Å from any heavy atom of the partner protein. Using the 1,113 compounds that commonly docked to the PPI sites, for each PPI site we computed a ranked list of the 1,113 compounds according to their binding scores. Then, for all pairs of PPI sites, we computed the sum of the Euclidean distance of the ranks of the same compounds. The drug ranking distance was then defined as the Z-score using the score distribution from all the pairs of PPI sites. If two PPI sites have similar compound docking results, it has a small negative Z-score. Thus, for all three metrics including PPI Score, a small value means that the PPI sites are similar to each other, and a large value indicates that the PPI sites are different.

Since PPI-Surfer compares PPI surfaces locally by surface patch characteristics rather than globally, it is expected that the program could yield PPI pairs that share locally similar physicochemical surface properties that may not be identified by the global similarity search, as we observed in our protein local surface-based binding site comparison methods, PatchSurfer (Sael and Kihara, 2010b; Chikhi et al., 2011; Sael and Kihara, 2012; Zhu et al., 2015; Zhu et al., 2016) and PL-PatchSurfer (Hu et al., 2014; Shin et al., 2016; Chiba et al., 2017; Shin and Kihara, 2018; Chiba et al., 2019; Shin and Kihara, 2019). To find out the unique characteristics of PPI Score, we analyzed the Pearson’s correlation between PPI Score and the other two reference metrics and hierarchical clustering based on the three metrics.

**FIGURE 3:**
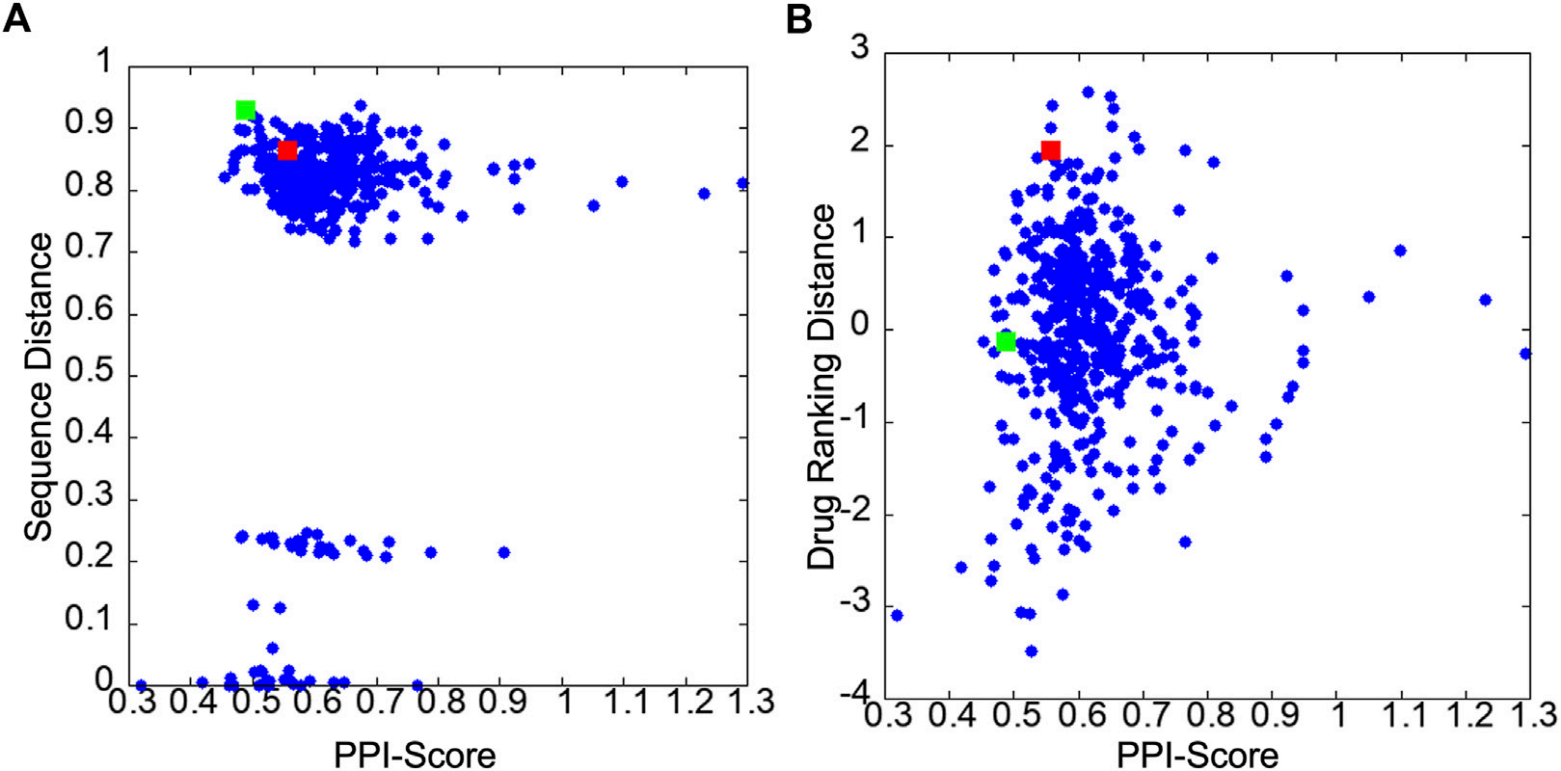
Score distribution of PPI Score of 32 PPIs in the 2P2I database. **(A)** scatter plot of the PPI Score and the sequence distance. **(B)** scatter plot of the PPI Score and the drug ranking distance. The red and green squares are 2FLUXP/1YCRAB and 1TUEAB/4GQ6AB pairs, which are discussed in the text.

Figures 3A, B show correlations between the PPI Score with the sequence distance and the drug ranking distance, respectively. In Figure 3A, pairs of PPI sites with a small sequence distance of less than 0.3 belong to the same protein families. For example, HRAS and KRAS has a sequence distance of 0.006 and X-linked inhibitor of apoptosis protein (XIAP) and cellular inhibitor of apoptosis 1 (CIAP) has a sequence distance of 0.22. Comparing PPI Score with the two reference metrics, PPI Score did not show overall strong correlation with the two metrics. The Pearson correlation coefficient with the sequence distance was 0.171, while it was 0.067 with the docking ranking distance. The overall small correlation coefficient between the sequence distance, the drug ranking score with PPI Score are not surprising because the sequence distance concerns the global similarity of the proteins while the PPI Score is local physicochemical similarity of PPI regions. Also, the drug ranking distance only indirectly reflects chemical similarity of local regions in PPIs sites. However, at the same time we can see cases that these metrics are related. In Figure 3A, very small PPI Scores only occurred for PPIs from the same family and reversely, relatively large PPI Scores (e.g., over 1) were only observed for PPIs from different protein families. Similarly, in Figure 3B, we see that a relatively small drug ranking distance was observed only when PPIs have a small PPI Score.

**FIGURE 4:**
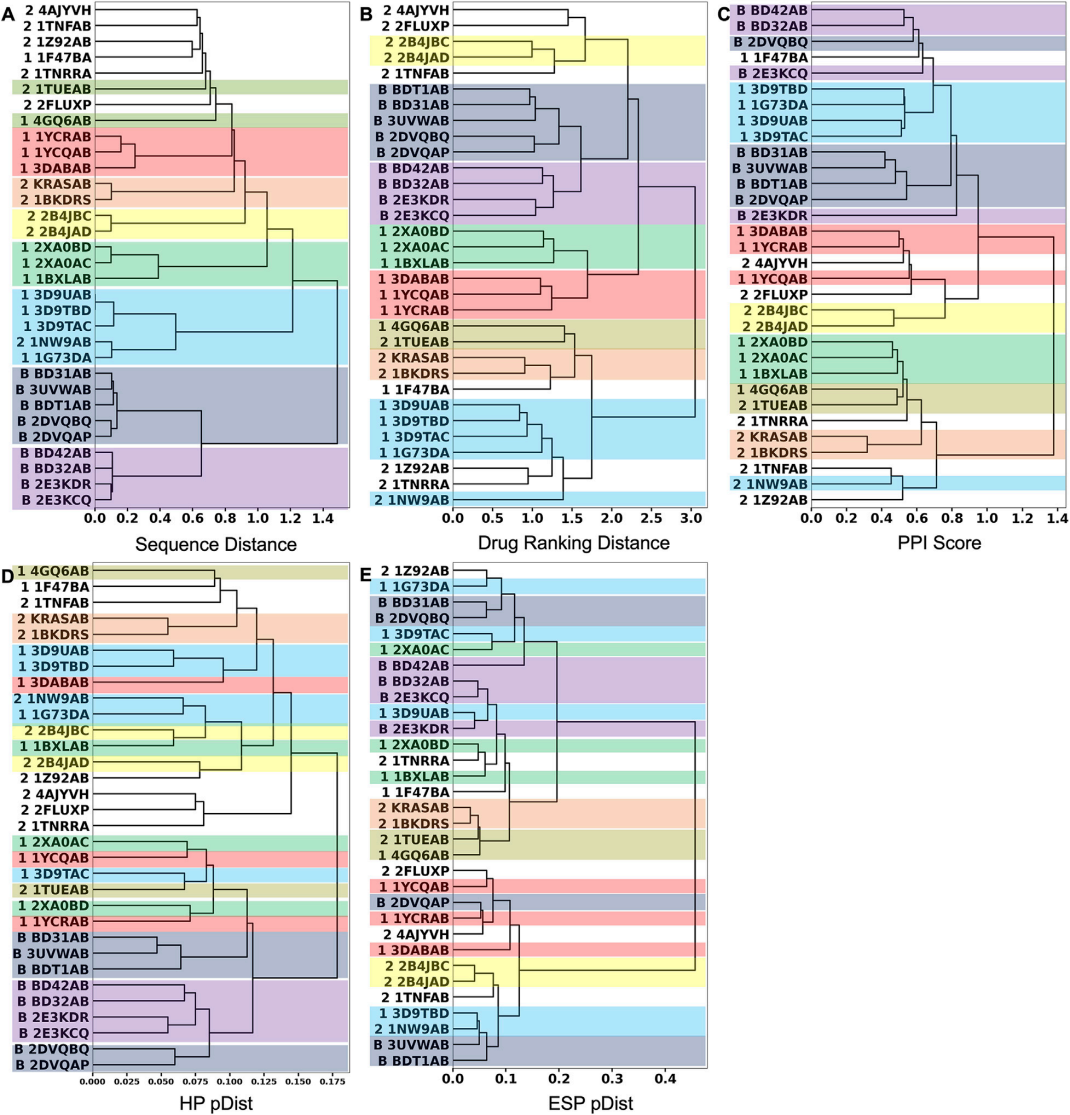
Hierarchical clustering of the 32 PPIs in the 2P2I database. We used the Ward’s clustering method. **(A)** clustering results using the sequence distance. **(B)** Clustering results using the drug ranking distance. **(C)** Clustering results using the PPI Score. **(D)** Clustering results using only the hydrophobicity term to quantify patch similarity in the PPI Score. After the corresponding patch pairs were identified between two PPIs, only the hydrophobicity term was used to compute *pDist* score (Eq. 6). **(E)** Clustering result using only the electrostatic potential term in *pDist*. In the dendrogram, the 2P2I class of each PPI is denoted in front of the PDB ID as follows: 1 for protein-peptide, 2 for protein-protein, and B for bromodomain. Entries in the same cluster by the sequence-distance are shown in the same color across the three dendrograms.

Next, we performed hierarchical clustering of PPI sites using the three metrics. Figures 4A–C show clustering results using the sequence distance, drug ranking distance, and the PPI Score, respectively.

We first discuss the clustering results using the two reference scores, the sequence distance (Figure 4A) and the drug ranking distance (Figure 4B). In these two clustering results, PPI sites of bromodomain-histone complexes, which are indicated with “B” in their IDs, were consistently grouped (shown in dark blue and violet boxes). They are separated into two subgroups, one colored in dark blue and the other in violet. Bromodomain-containing proteins have two bromodomains in their structures. The five members of the dark blue box are the first bromodomains (BD1s) and all four members of the violet box are the second bromodomains (BD2s). PPI sites of these two groups are slightly different in the histone peptide binding region. The binding site near the C-terminal of the co-complexed histone peptide is different between BD1s and BD2s. BD2s form a helix, while BD1s have a coil structure.

Besides the bromodomain entries, all the clusters formed by the sequence distance (Figure 4A) within 0.6 are consistent with clusters in the dendrogram by the drug-ranking distance (Figure 4B). A cluster of five PPI sites (sky blue box), four PPI sites of CIAP (3D9UAB, 3D9TBD, 1G73DA, and 3D9TAC) and XIAP (1G73DA) in the protein-peptide complex class (indicated with “1” at their IDs) and one PPI site, XIAP (1NW9AB) from the protein-protein complex class (indicated with “2” at their IDs), are grouped in both dendrograms although additional entries are included in the drug ranking distance dendrogram (sky blue). Despite residing in different classes, 1G73DA and 1NW9AB are both XIAP proteins with different binding partners, which explains why they were grouped in the sequence distance dendrogram. Similarly, clusters indicated with boxes in different colors (green, yellow, orange, and red) are consistent between the two dendrograms.

Although the sequence-based clustering (Figure 4A) and the drug ranking-based clustering (Figure 4B) are consistent in many places, there are also differences. In the sequence-distance-based dendrogram (Figure 4A), a loose cluster with a distance over 0.6, which includes eight PPI sites from the protein-peptide complex and protein-protein complex classes, was formed at the top of the dendrogram. But these PPI sites are scattered in the drug-ranking distance-based dendrogram, indicating that the sequence distance at 0.6 or lower is not generally sufficient to assume similar drug-binding properties.

Next, we examine the clustering results based on the PPI Score (Figure 4C) relative to the two reference metrics. As visualized in colors, most of the clusters in the sequence- and drug-ranking-based dendrograms were consistently observed in the PPI Score-based clustering results. The common clusters include four out of five entries in the bromodomain, BD1 (dark blue), three out of four in the bromodomain, BD2 (violet), four out of five in the CIAP and XIAP complexes (sky blue), B-cell lymphoma (green), HIV integrase (yellow), KRAS and human RAS proteins (orange), and two out of three entries of double mutant 2 proteins (red). The average PPI Score of PPI pairs from same family with a (sequence distance less than 0.3 was 0.574.

On the other hand, there are also differences. In the PPI Score-based clustering, two subgroups of bromodomain cluster (dark blue and violet) were separated in the dendrogram and some entries were isolated from the rest of the subgroup members. Among five BD1 PPI sites, 2DVQBQ was not clustered together and placed close to the BD2 cluster (violet). The main reason for this separation turned out to be the number of patches, i.e., the size of the PPI interface. The PPI Score (Eq. 8) includes a term for PPI site size difference, which accounts for the differences of number of patches. As 2DVQBQ has a 17-residue-long histone peptide in the complex, which is longer than peptides of the other four BD1 members (peptides of BD31AB, 3UVWAB, BDT1AB are13 residue long 2DVQAP has a 10-residues-long peptide), the PPI site of 2DVQBQ had more surface patches than the other members. The PPI site of 2DVQBQ consists of 20 patches, while the other members had 13–16 patches (average: 14.5) (Figure 2). This patch number difference of 2DVQBQ made the SD term larger to the rest of the BD1 members, which resulted in the separation in the dendrogram (Figure 4C). Besides the SD term in Eq. 8, 2DVQBQ has similarities to other BD1 members in other terms. For example, 2DVQBQ and BD31AB has an average physicochemical similarity of patches, *pDist* (the first term in Eq. 8) of 0.268, the first term of PPI Score, which is the seventh rank of *pDist* among all pairs of 31 PPI sites.

The PPI site size difference in the complexes also explains why 2E3KDR was separated far from two clustered BD2 members, BD42AB and BD32AB (violet). The length of the 2E3KDR histone peptide is 14, shorter than 16 that bind to the other BD2 PPIs. Moreover, the C-terminal tail of the 2E3KDR histone does not bind to the bromodomain, which resulted in only four PPI patches for 2E3KDR. This is much smaller than the average number of patches of the other three BD2s, 16.3, which largely influenced to the PPI Score between 2E3KDR and the rest.

**FIGURE 5:**
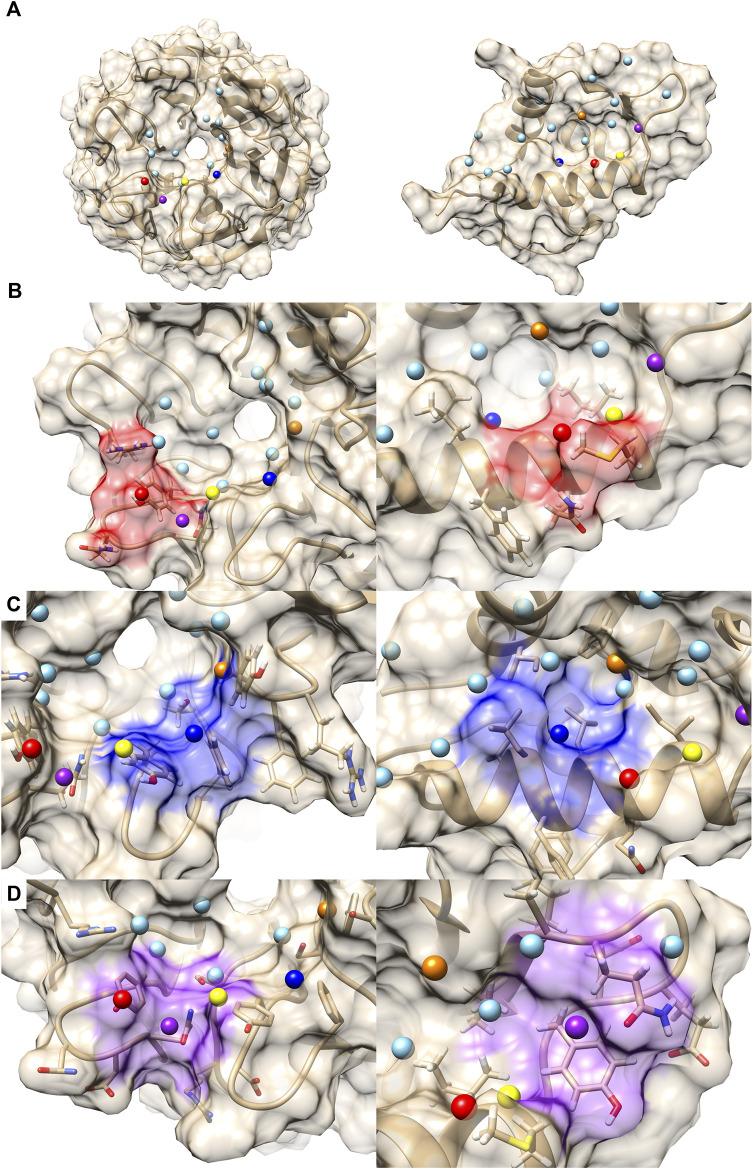
Comparison with 2FLUXP and 1YCRAB, which are clustered in the PPI Score-based clustering. **(A)** Molecular surfaces of KEAP1 (2P2I ID: 2FLUXP, left) and MDM2 (2P2I ID: 1YCRAB, right) and seed points (center of patches, sky blue sphere). The seed points of patch pairs identified by PPI-Surfer are colored in red, blue, purple, orange, and yellow. The pairs have the same color codes. **(B–D)** Seed points of the patch pairs and molecular surface regions that are closer than 6 Å from the seed points. Patch #11 of KEAP and #6 of MDM2 red, panel **(B)**, patch #3 of KEAP and #5 of MDM2 blue, panel **(C)**, and patch #13 of KEAP and #11 of MDM2 purple, panel **(D)**. The KEAP patches are shown on the left and MDM2 patches are shown on the right.

The next example of difference observed in the PPI Score-based clustering is for three PPIs of double mutant 2 proteins (red). In the PPI Score-based clustering, the cluster of double mutant 2 proteins included two additional PPI sites, von Hippel-Lindau disease tumor suppressor (VHL, 2P2I ID: 4AJYVH) and Kelch-like ECH-associated protein 1 (KEAP1, 2P2I ID: 2FLUXP). 2FLUXP has a β-propeller structure, complexed with a 16-residue-long peptide with two β-strands. Figure 5A shows PDB structures of 2FLUXP and 1YCRAB (mouse double minute 2 protein (MDM2) complexed with p53). This PPI site pair had distances of 0.556, 0.863, and 1.943 for PPI Score, sequence similarity score, and docking ranking score, respectively (red square in Figures 3A, B); thus, these two PPIs are close in terms of PPI Score despite the larger distance between them in terms of the sequence similarity and the drug ranking score. The PPI Score of 0.556 is smaller than 0.574, which is the average PPI Score value of PPIs from the same protein family.

A closer look at their PPI sites indeed identifies similarities in corresponding surface patches. The top five patch pairs identified by PPI-Surfer are shown in Figure 5A. Patch #11 of 2FLUXP and patch #6 of 1YCRAB (red-colored patches, Figure 5B) have polar residues such as TYR and GLN. Another patch pair, 2FLUXP #3 and 1YCRAB #5 (blue-colored patches, Figure 5C), are mainly composed of hydrophobic residues, TYR, PHE, and SER for 2FLUXP #3 and LEU, ILE, and PHE for 1YCRAB #5. Similarly, both 2FLUXP #13 and 1YCRAB #11 (purple-colored patches, Figure 5D) contain polar (GLN), negative (ASP), and aromatic residues (TYR). The patches in the two PPIs are also numerically close. The matched patch pairs have high physico-chemical similarities, especially for electrostatic potential and hydrophobicity. The average 3DZD distance of electrostatic potential was 0.07, which is less than or comparable to double mutant protein PPI pairs (0.09 for 3DABAB-1YCRAB, 0.06 for 1YCRAB-1YCQAB, and 0.10 for 1YCQAB-3DABAB). Similarly, average hydrophobicity distance of 2FLUXP-1YCRAB was 0.10, while the three pairs of the red cluster have 0.09, 0.07, and 0.08.

**FIGURE 6:**
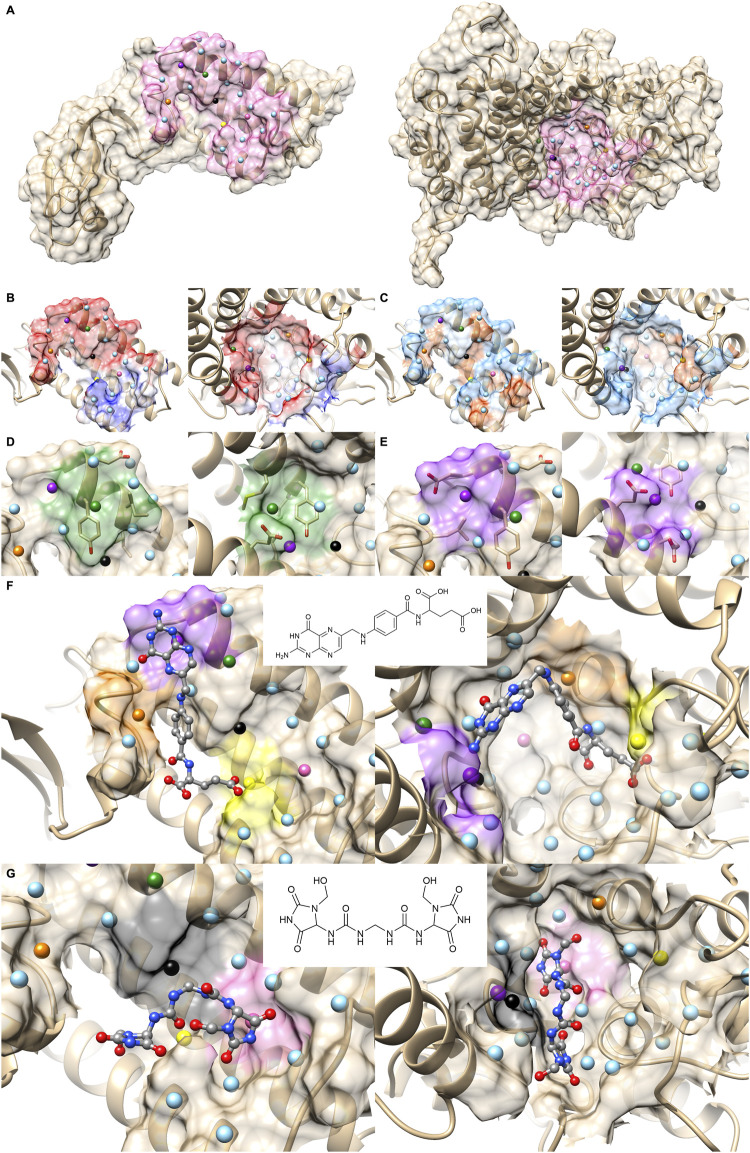
Comparison with 1TUEAB and 4GQ6AB, which are clustered in the PPI Score-based clustering. **(A)** Molecular surfaces of replication protein E1 (2P2I ID: 1TUEAB, left) and menin (2P2I ID: 4GQ6AB, right) and seed points (center of patches, sky blue sphere). The interface extracted is highlighted as pink. The seed points of corresponding patch pairs identified by PPI-Surfer are colored in green, purple, orange, yellow, black, and pink. **(B)** The electrostatic potential mapped on the PPI surface. The color changes from red (negative) to blue (positive). **(C)** Hydrophobicity mapped on the PPI surface. The color changes from sky blue (hydrophilic) to orange (hydrophobic). **(D)** Patch pairs that have the closest distance from 1TUEAB and 4GQ6AB. 1TUEAB patch #8 and 4GQ6AB patch #21. The seed points are shown in green. Surface regions within 6 Å to the seed points are colored also in green. **(E)** Patch pairs that have the second closest distance from 1TUEAB and 4GQ6AB. 1TUEAB patch #12 and 4GQ6AB patch #22. The seeds and the surface regions are colored in purple. **(F, G)** Predicted binding poses of folic acid panel **(F)** and imidazolidinyl urea panel **(G)** on the PPI surfaces of 1TUEAB and 4GQ6AB. Two-dimensional structures of the compounds are shown at the middle of the panels.

Figure 6 is a case where two PPI sites were similar and clustered by the drug ranking score (Figure 4B) and PPI-score (Figure 4C), but not by the sequence similarity (Figure 4A). They are protein E1 (2P2I ID: 1TUEAB) and menin (2P2I ID: 4GQ6AB) (olive in Figure 4). PPI Score of these PPIs is 0.487, closer than the average PPI Score of the same protein family. Their sequence, drug ranking, and PPI Score-based distances are shown in green square in Figures 3A, B. These two proteins do not share a high sequence and structural similarities (sequence identity: 4.3% (Needleman and Wunsch, 1970), TM-Score structural similarity: 0.16 (Zhang and Skolnick, 2004)). Figure 6A shows their overall structures with the PPI sites highlighted in pink and seed points of surface patches shown by dots. The patch pairs identified by PPI-Surfer are colored in green, purple, orange, yellow, black, and pink. The structures of their partner proteins are also different; replication protein E1 binds to regulatory protein E2 with 218 amino acids, while menin binds to a 12-residue-long peptide. Despite these differences, 1TUEAB and 4GQ6AB have similar PPI sites. The PPI of 1TUEAB and 4GQ6AB consists of 22 and 24 patches, thus they are similar in size. Also, as shown in Figures 6B, C, these two PPIs are at a cavity of the protein surface and have a somewhat similar distribution of the electrostatic potential and hydrophobicity. The average 3DZD distances of electrostatic potential and hydrophobicity are 0.05 and 0.10, respectively, which are smaller or comparable to the same protein family as observed in double mutant 2 protein family. The two PPIs are mostly negatively charged (colored in red in Figure 6B) and hydrophobic regions (colored in orange in Figure 6C) locate at the boundary of the PPIs. In Figure 6D, we closely looked at patch pairs 1TUEAB #8 and 4GQ6AB #21 (green seed points), which have the closest distance among corresponding pairs. The patch #8 of 1TUEAB is surrounded by hydrophobic residues (ILE), a negatively charged residue (GLU), and an aromatic residue (TYR) and similarly, the patch #21 in 4GQ6AB is also composed of GLU, TYR, ILE, and MET. The second closest patch pair (Figure 6E) was1TUEAB #12 and 4GQ6AB #22 (purple seed points). They are both composed of a charged residue (GLU), a hydrophobic residue (LEU), and an aromatic residue (TYR).

In terms of the drug ranking distance, 1TUEAB and 4GQ6AB have four compounds in common among their top 10 scoring docked compounds. Among the four common compounds, we can see two compounds have similar binding modes on these two PPI sites. First one show in Figure 6F is folic acid. The heterobicyclic ring is close to the purple patch in both PPI sites, which has negative electrostatic potential and hydrophilic character, and the benzyl ring in the middle of the compound was placed close to the orange patch composed of a negatively charged surface with half hydrophobic and half hydrophilic character. Also, the terminal carboxyl group was predicted to bind to the yellow patch, which is hydrophilic. Another common compound ranked with in top 10 for the two PPI was imidazolidinyl urea (Figure 6G). When surface patches that are closer than 5 Å from the docked pose on both PPIs are examined, the black patch that has negative and hydrophobic character is located close to the two peptide bonds, and one of the five-membered heterocyclic ring is close to the pink patch, which has neutral electrostatic potential and hydrophobic.

For comparison with the PPI Score, we only used the hydrophobicity term and the electrostatic term to define the patch distance, *pDist* (Eq. 6) in Figures 4D, E, respectively. Clusters made by the single term made showed less consistent results with the two reference scores (Figures 4A, B) as well as by the full PPI Score (Figure 4C).

To summarize, PPI Score did not show a strong overall numerical correlation to two existing reference metrics, the sequence distance and the drug ranking distance. However, it holds general consistency with the two metrics in identifying similarity in PPI pairs. PPIs of the same protein family have a small PPI distance. Also, a small drug-ranking distance corresponds to a small PPI Score. Therefore, clustering results of PPIs with PPI Score included many common clusters with the results by the two reference metrics as shown in Figure 4. There are differences, of course, which can be explained by a close look. PPI Score can be large (more distance) if two PPI sites have different size and identified similar PPIs by PPI Score have similarity in the electrostatic potential and hydrophobicity.

### 3.2 Identifying PPI hotspots

As PPI-Surfer compares two PPI interfaces locally and identifies similar local patches, it could be useful to identify interface hotspots. The hotspots are residues at a PPI interface and are responsible for maintaining a high binding affinity of the PPI. Therefore, hotspot residues are often preserved both sequentially and spatially among related proteins (Glaser et al., 2003; Ma et al., 2003; Aytuna et al., 2005; Res and Lichtarge, 2005). In this section, we tested PPI-Surfer on three pairs of protein complexes that have common hotspot residues. The hotspot residues were defined by the MAPPIS method, which superimposes the structures of interacting protein pairs and identifies residues that have similar physico-chemical interactions between them and defines them as hotspot residues (Shulman-Peleg et al., 2007). In the paper of MAPPIS, the authors collected 71 PDB entries from 14 proteins and provided hotspot information for three protein sets. They are sets of ribonucleases inhibitor complexes (four PPIs), immunity proteins with colicin DNase (six PPIs), and T-cell receptors (TCR) with superantigens (six PPIs) (Table 1 of Ref. 17). Each set contains different protein complexes but they often share the same proteins with 100% identical sequences. Thus, here, we ran PPI-Surfer for pairs of proteins that are not 100% identical.

**TABLE 1:** Detection of hotspots using PPI-Surfer. Three pairs of PPI surfaces were compared with PPI-Surfer and corresponding patches were identified between them. Among the identified patch pairs, hotspot patches and residues were reported. Under each PDB ID, we listed the number of hotspot patches and hotspot residues of the entry. The numbers of detected hotspot patches and residues by PPI-Surfer are shown in the column of Identified.

Ribonucleases			
PDB ID	1a4yA	1dfjB	Identified
Hotspot Patches	7	12	6
Hotspot Residues	3	3	3
Colicin Immunity Proteins			
PDB ID	1bxiA	1znvA	Identified
Hotspot Patches	12	14	9
Hotspot Residues	5	5	5
T-Cell Receptors			
PDB ID	1jckA	1ktkE	Identified
Hotspot Patches	10	12	8
Hotspot Residues	4	4	4

In the first set, we compared ribonuclease inhibitors (RIs) from the PDB entry 1a4y and from 1dfj. The sequence identity between them is 77%. Three hotspot residues in RI have been experimentally confirmed, which are TYR434, ASP435, and TYR437 (the residue numbers are from 1a4y A chain). In order to evaluate the performance of PPI-Surfer on hotspot identification, we defined a hotspot patch as a patch that locates closer than 5 Å to any heavy atom of a hotspot residue when computed from its seed point. For example, seven hotspot patches were identified on RI with angiogenin (1a4yA).

**FIGURE 7:**
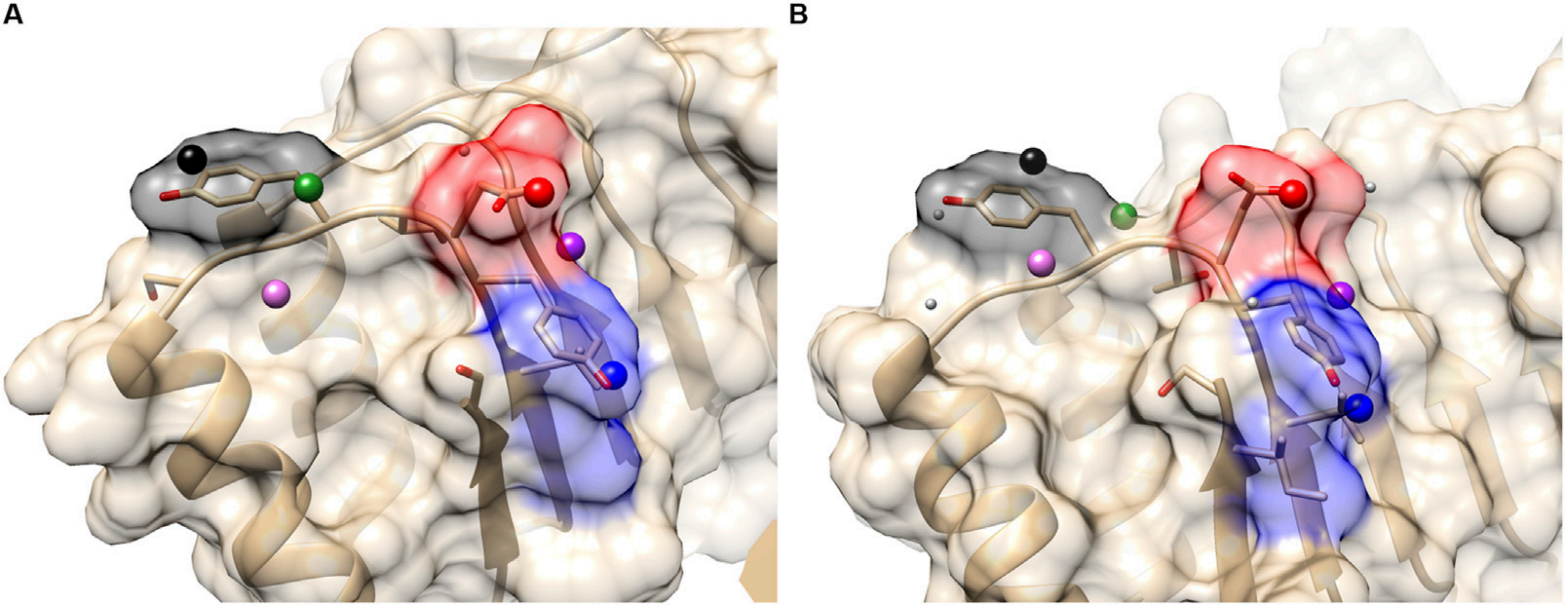
Hotspot patches in two ribonuclease inhibitors (RIs), which were identified by PPI-Surfer. **(A)**, 1a4yA. Three hotspot residues, TYR434, ASP435, and TYR437 are shown in the stick representation. **(B)**, 1dfjB. The three hotspot residues, TYR430, ASP431, and TYR433 are shown in the stick representation. These two receptors have a sequence identity of 77%. The seed points of six corresponding patch pairs identified by PPI-Surfer are shown in spheres in the same colors. In addition, other seeds of hot spot patches are shown with light gray spheres. The three lowest *pDist* patch pairs and their surfaces are colored black, red, and blue.

Table 1 summarizes the results of hotspot patch and residue detection by PPI-Surfer. Hotspot residues and patches of RIs are shown in Figure 7 as an example. Out of the seven hotspot patches on the PPI interface of 1a4yA (Figure 7A), six of them are identified as common when compared to 1dfjB (Figure 7B). All the six patches are located near experimentally determined hotspots (TYR434, ASP435, and TYR437 in 1a4yA). In Figure 7, the three lowest *pDist* distance (i.e., the three most similar) patches are colored in black, red, and blue, which distribute among all three hotspot residues.

The second pair is proteins from the immunity proteins with colicin DNases, 1bxiA and 1znvA, which have a sequence identity of 59%. GLU30, ASP51, TYR54, TYR55, and PRO56 in 1bxiA are hotspot residues that were identified by experiments. MAPPIS identified all these five residues (Shulman-Peleg et al., 2007). Likewise, PPI-Surfer identified the five residues in 1bxiA when compared with 1znvA. The last pair is 1jckA and 1ktkE, from T-cell receptors. The sequence identity between them is 55%. They have four experimentally identified hotspot residues, ALA52, GLU53, THR55, and PRO70 (the residue numbers are from 1jckA). PPI-Surfer was able to identify hotspot patches that correspond to all four hotspot residues.

The results on this experiment show that PPI-Surfer detects hotspot patches since they are similar in physico-chemical properties among related proteins. The difference between MAPPIS and PPI-Surfer is that while MAPPIS takes protein complexes as input and identifies common interactions through structural alignments, PPI-Surfer identifies similar regions without the information of the binding partner, thus without structural alignment operation.

### 3.3 Predicting SARS-CoV-2 virus protein inhibitor

**FIGURE 8:**
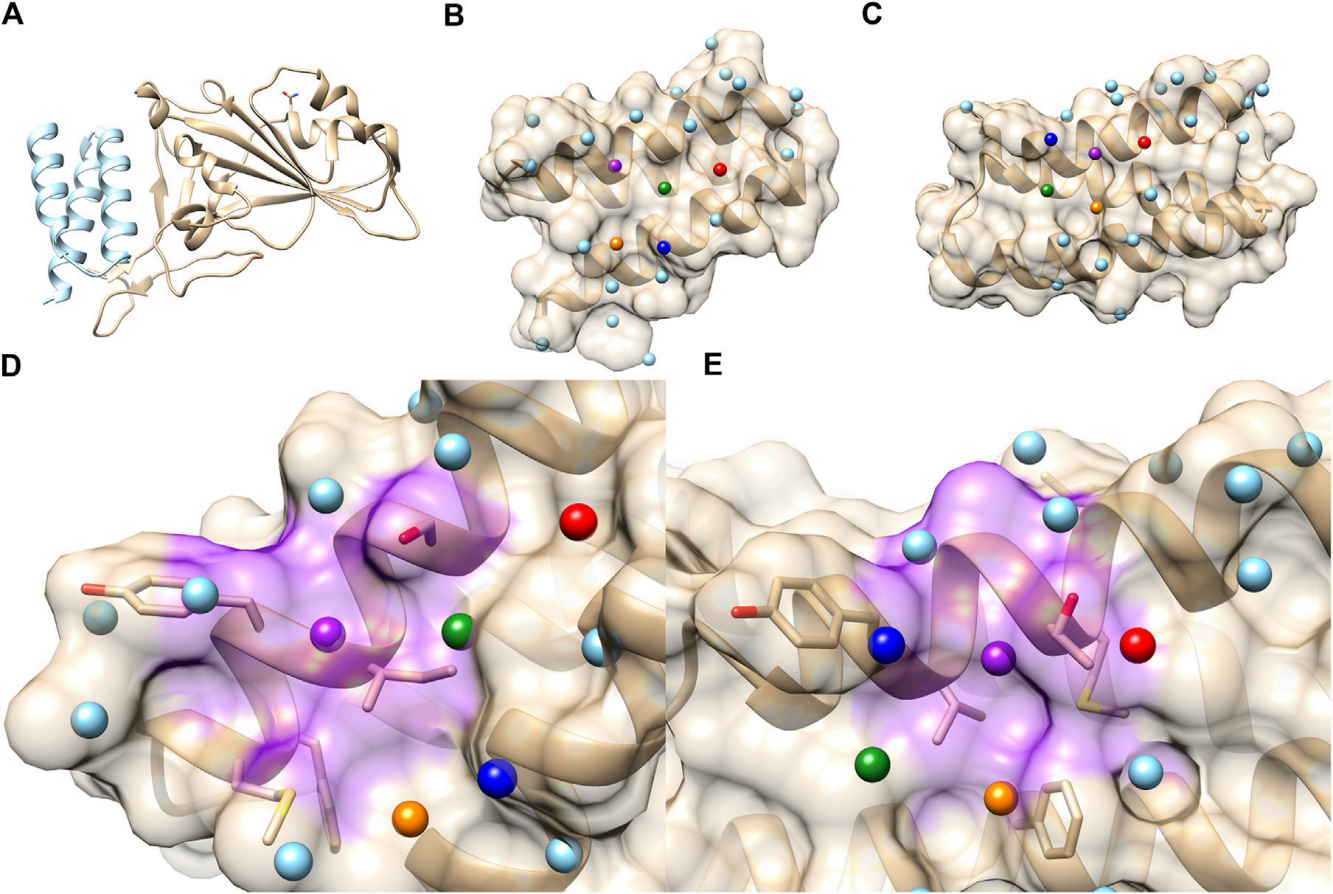
PPI sites of LCB1 and LCB3. **(A)** The crystal structure of the ACE2 RBD domain (Gold) and LCB1 (sky blue). PDB ID: 7JZU. **(B)** The structure and seed points (spheres) of LCB1. **(C)** The structure and seed points (spheres) of LCB3. Top 5 closest patch pairs are indicated by the same color. **(D, E)** The closest patch pairs (purple) from **(D)** (LCB1 #20) and **(E)** (LCB3 #12). The residues surrounding LCB1 #20 (ILE, SER, TYR, MET, and PHE) and LCB3 #12 (LEU, MET, THR, TYR, and PHE) are shown in a stick representation.

Next, we applied PPI-Surfer to a dataset of SARS-CoV-2 mini protein inhibitors, a set of helical proteins that were computationally designed to bind to the receptor-binding domain (RBD) of human Angiotensin Converting Enzyme 2 (ACE2). The bound mini protein inhibitor interferes with the interaction of RBD with the SARS-CoV-2 spike protein (Cao et al., 2020). Many mini-protein inhibitors were initially computationally designed and then their interactions were verified by experiments. Through this process, the authors identified eight proteins, named LCB1 to LCB8, which bind to RBD with a dissociation constant, K_D_, of 1–20 nM (Cao et al., 2020). The amino acid sequence lengths of the eight proteins are between 56 and 65. The global pairwise sequence identities of the eight LCB peptides ranged from 7.6% to 50.0% (Table 2). As shown in the table, LCB2 and LCB7 have a sequence identity of 50% between each other and the rest of the pairs share less than 30% identity. The complex structures with ACE2 were made available in PDB only for LCB1 (PDB ID: 7JZU, Figure 8A) and LCB3 (PDB ID: 7JZM). Additionally, we modeled the tertiary structure of the remaining six designed inhibitors, LCB2, 4, 5, 6, 7, 8, using GalaxyTBM (Ko et al., 2012) and then docked them to RBD using GalaxyTongDock (Park et al., 2019). All the designed proteins were predicted to have helix bundle fold. The binding residues information of ACE2, which is available in Supplementary Table S1 of Cao et al. (2020), was used to guide protein-protein docking.

**TABLE 2:** Pairwise sequence identities (%) between LCBs.

	LCB2	LCB3	LCB4	LCB5	LCB6	LCB7	LCB8
LCB1	27.1	18.1	19.7	26.4	23.3	28.3	24.6
LCB2		29.4	23.4	7.6	25.9	50.0	17.6
LCB3			19.3	11.2	24.6	24.6	19.0
LCB4				18.6	21.7	20.0	23.0
LCB5					7.7	23.3	14.9
LCB6						25.8	12.4
LCB7							10.8

Starting from each of the eight binders, we constructed decoy proteins by sequence optimization using Rosetta FixBB (Kuhlman et al., 2003), where the sequence of each starting LCB was mutated randomly while maintaining the backbone conformation in the docking pose with RBD. Among those generated sequences, we selected 10 amino acid sequences which have the 10 lowest Rosetta energy. Thus, for each LCB, 10 mutant proteins that share the same backbone structure and PPI interface were produced. The predicted structures and their mutants are made available at a public repository, doi: 10.5281/zenodo.7214132.

On the library of the eight designed binders and their decoys, we calculated the surface similarities among their PPIs using PPI-Surfer and examined if the PPI Score can discriminate the binders from other generated decoy proteins. We first compared LCB1, LCB3, and their decoys in terms of the PPI Score. We started with these two binders because LCB1 and LCB3 has a low sequence identity of 18.3% and both have experimentally determined complex structures with RBD. We performed PPI site comparison in two directions. First, the PPI site of LCB1 was compared with the PPI site of LCB3 and its 10 decoys. Next, we computed the PPI site similarity from LCB3 with LCB1 and its 10 decoys.

As shown in Table 3, when LCB1 was compared with LCB3 and its decoys, PPI-Surfer identified that the binder, LCB3, is the most similar PPI site to LCB1 than the other 10 decoys (the left column) with a substantial Z-score of less than −2. Similarly, the search from LCB3 against LCB1 and its 10 decoys also identified LCB1 as the most similar PPI site. Thus, PPI-Surfer was able to identify the binder pairs from decoys despite their low sequence identity. In Figures 8B, C, we showed top five corresponding surface patches from PPI sites of LCB1 and LCB3 in the same colors. All the corresponding patches are located around the center of the RBD binding site, which is between two helices. The most similar patch pair (shown in purple in Figures 8D, E) has hydrophobic characteristics. The seed point of LCB1 patch #20 (Figure 8D) is surrounded with ILE, SER, TYR, MET, and PHE, and the partner patch from LCB3 #12 (Figure 8E) is composed of LEU, MET, THR, TYR, and PHE.

**TABLE 3:** PPI Scores between ACE2 binders, LCB1, LCB2 and their mutants (decoys). The numbers in the parentheses are Z-Scores.

Compared with LCB1 (PDB ID: 7JZU)		Compared with LCB3 (PDB ID: 7JZM)	
Protein	PPI Score	Protein	PPI Score
LCB3	0.877 (−2.101)	LCB1	0.877 (−2.070)
Mutant #1	1.024 (0.657)	Mutant #1	0.941 (−0.449)
Mutant #2	1.068 (1.482)	Mutant #2	0.995 (0.919)
Mutant #3	1.005 (0.300)	Mutant #3	0.985 (0.665)
Mutant #4	0.945 (−0.825)	Mutant #4	0.976 (0.437)
Mutant #5	1.010 (0.394)	Mutant #5	0.934 (−0.626)
Mutant #6	0.977 (−0.225)	Mutant #6	0.987 (0.716)
Mutant #7	0.976 (−0.244)	Mutant #7	1.006 (1.197)
Mutant #8	1.055 (1.238)	Mutant #8	0.951 (−0.196)
Mutant #9	0.956 (−0.619)	Mutant #9	0.981 (0.564)
Mutant #10	0.986 (−0.056)	Mutant #10	0.913 (−1.158)

We further analyzed PPI sites of other six LCBs, LCB2, 4, 5, 6, 7, and 8. We compared the PPIs of the LCBs and their decoys with LCB1 and LCB3, the two binders that have crystal structures, in terms of the PPI Score by PPI-Surfer. To rank the PPIs, first, we computed Z-Scores of PPI Scores from LCB1 and LCB3 separately and averaged them. In Figure 8, the averaged Z-score of PPI Score are shown relative to the Rosetta energy.

**FIGURE 9:**
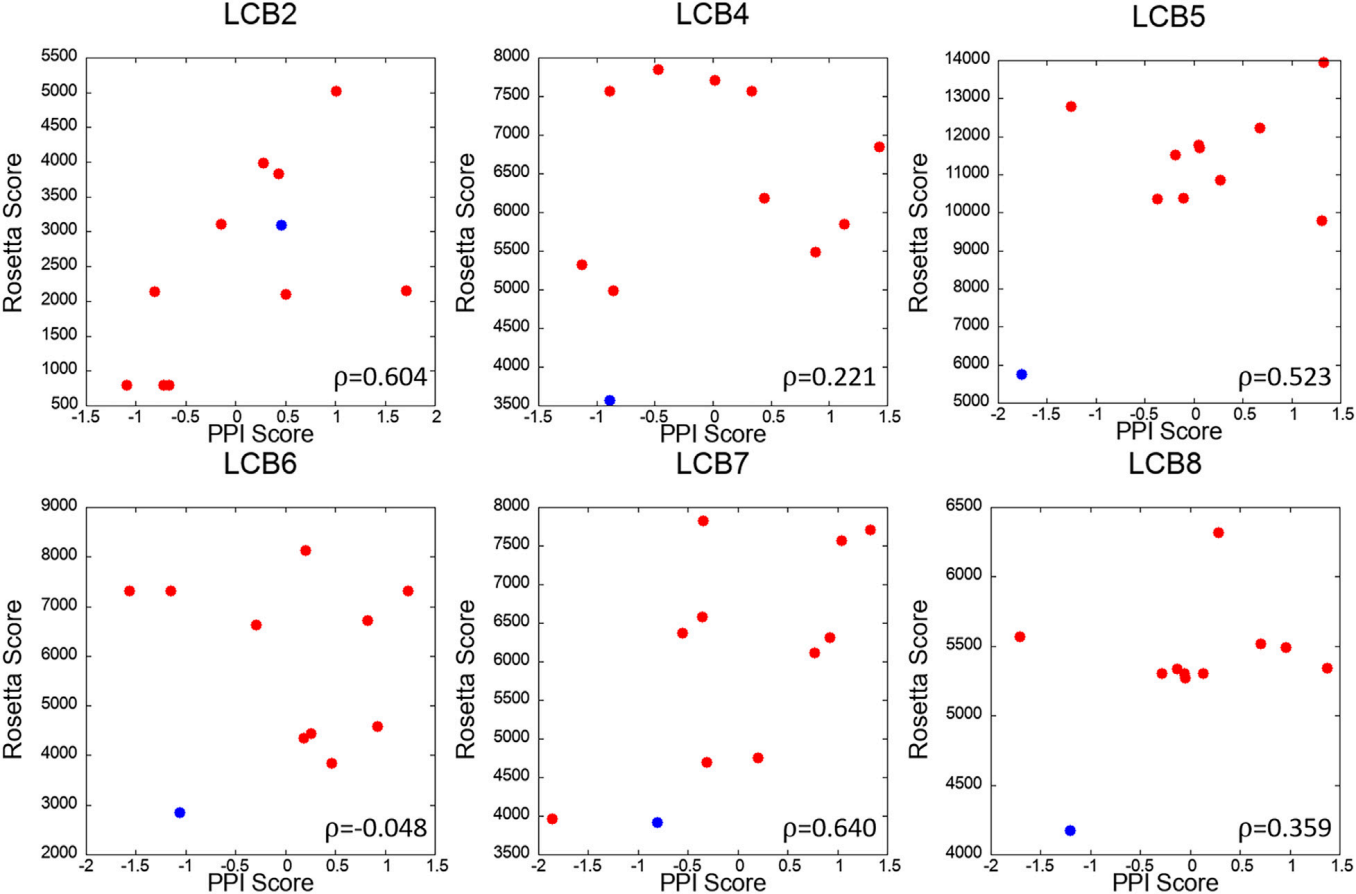
Correlation of PPI Score and the Rosetta binding energy for each LCB and its decoys. The *x*-axis is the average Z-scores of PPI Scores (computed from LCB1 and LCB3), and the *y*-axis is Rosetta score of the complex. The red dots are 10 mutants generated by Rosetta and the blue dot is the starting binder. Pearson’s correlation coefficient of each scatter plot is written the lower right corner of the plot.

The PPI-Surfer identified the original sequence of LCB5 as the top. Besides the original sequence of LCB was the second closest PPIs with LCB1 and LCB3 for three other cases (LCB4, 6, 7) (Figure 9). LCB5 was the fourth 4^th^ strongest binder among the 8 LCBs and prevented ∼20% of virus infections when ∼100 nM was treated (Cao et al., 2020). By the authors’ experiment, LCB4 showed a nanomolar range of EC_50_ (Cao et al., 2020). LCB2 is an interesting case. PPI Score did not identify the original LCB2 among the closest PPI to LCB1 and 3, but it recognized decoys that have the lowest Rosetta energy as the closest PPI. PPI Score and the Rosetta energy showed substantial correlation of 0.604.

To summarize, PPI-Surfer identified experimentally verified binders or the one that has the lowest binding energy as the closest PPIs by the PPI surface similarity, which is not obvious from the sequence similarity of the proteins.

## 4 Conclusion

We developed PPI-Surfer, which quantifies PPI surface similarity that considers five physicochemical properties relevant for forming PPIs. A strength of PPI-Surfer is it can identify similar local regions in given PPIs, unlike existing methods that compares predefined PPI regions. PPI-Surfer is unique in that it identifies similarity of surface properties that do not rely on amino acid sequence and atom positions in the 3D structure of proteins. When tested on the 2P2I database, we found that the PPI Score by PPI-Surfer is in general in a good agreement with the protein sequence distance and the drug ranking distance yet showed interesting cases where PPI-Surfer uniquely identified surface similarity that are not obvious from the sequence distance. PPI-Surfer was also able to identify hotspots in PPIs. When tested on the SARS-CoV-2 protein inhibitors, PPI-Surfer was able to find surface similarities of potent binders that again do not have a high sequence similarity.

At this junction, we acknowledge the limitations of this work. While the results presented are promising, the robustness of the method must be further tested on different data and tasks as the datasets used in this works are not large. Specifically, direct relevance of identified PPI interface similarity to PPI drugs has not been examined as the number of approved PPI drugs is too small. Additionally. PPI-Surfer does not take into account the flexibility of protein structures. As a result, potential drug binding sites in PPIs that appear in the dynamics (cryptic binding sites) are not detected.

Despite the importance and growing interests in PPI drugs, computational protocols for the development are not well established. PPI-Surfer would be a valuable tool for finding similarities and classifying PPI sites and aid protein-based PPI drug discovery.

## Data Availability

The original contributions presented in the study are included in the article, further inquiries can be directed to the corresponding author.

## References

[B1] Aderinwale T. Bharadwaj V. Christoffer C. Terashi G. Zhang Z. Jahandideh R. (2022). Real-time structure search and structure classification for AlphaFold protein models. Commun. Biol. 5, 316. 10.1038/s42003-022-03261-8 35383281 PMC8983703

[B2] Alzyoud L. Bryce R. A. Al Sorcky M. Atatreh N. Ghattas G. A. (2022). Structure-based assessment and druggability classification of protein–protein interaction sites. Sci. Rep. 12, 7975. 10.1038/s41598-022-12105-8 35562538 PMC9106675

[B3] Arkin M. R. Randal M. DeLano W. N. Hyde J. Luong T. N. Oslob J. D. (2003). Binding of small molecules to an adaptive protein-protein interface. Proc. Natl. Acad. Sci. U.S.A. 100, 1603–1608. 10.1073/pnas.252756299 12582206 PMC149879

[B4] Arkin M. R. Wells J. A. (2004). Small-molecule inhibitors of protein-protein interactions: Progressing towards the dream. Nat. Rev. Drug Discov. 3, 301–317. 10.1038/nrd1343 15060526

[B5] Aytuna A. S. Gursoy A. Keskin O. (2005). Prediction of protein-protein interactions by combining structure and sequence conservation in protein interfaces. Bioinformatics 21, 2850–2855. 10.1093/bioinformatics/bti443 15855251

[B6] Basse M. J. Betzi S. Morelli X. Roche P. (2016). 2P2Idb v2: Update of a structural database dedicated to orthosteric modulation of protein-protein interactions. Database 2016, baw007. 10.1093/database/baw007 26980515 PMC4792518

[B7] Budowski-Tal I. Kolodny R. Mandel-Gutfreund Y. (2018). A novel geometry-based approach to infer protein interface similarity. Sci. Rep. 8, 8192. 10.1038/s41598-018-26497-z 29844500 PMC5974305

[B8] Canterakis N. (1999). “3D Zernike moments and Zernike affine invariants for 3D image analysis and recognition,” in Proceedings of 11th Scandinavian Conference on Image Analysis, Kangerlussuaq, Greenland, June 7-11, 1999.

[B9] Cao L. Goreshnik I. Coventry B. Case J. B. Miller L. Kozodoy L. (2020). De novo design of picomolar SARS-CoV-2 miniprotein inhibitors. Science 370, 426–431. 10.1126/science.abd9909 32907861 PMC7857403

[B10] Cheng S. Zhang Y. Brooks C. L. (2015). PCalign: A method to quantify physicochemical similarity of protein-protein interfaces. BMC Bioinforma. 16, 33. 10.1186/s12859-015-0471-x PMC4339745 25638036

[B11] Cheng T. Zhao Y. Li X. Lin F. Xu Y. Zhang X. (2007). Computation of octanol-water partition coefficients by guiding an additive model with knowledge. J. Chem. Inf. Model. 47, 2140–2148. 10.1021/ci700257y 17985865

[B12] Chiba S. Ishida D. Ikeda K. Mochizuki M. Teramoto R. Taguchi Y. H. (2017). An iterative compound screening contest method for identifying target protein inhibitors using the tyrosine-protein kinase Yes. Sci. Rep. 7, 12038. 10.1038/s41598-017-10275-4 28931921 PMC5607274

[B13] Chiba S. Ohue M. Gryniukova A. Borysko B. Zozulya S. Yasuo N. (2019). A prospective compound screening contest identified broader inhibitors for Sirtuin 1. Sci. Rep. 9, 19585. 10.1038/s41598-019-55069-y 31863054 PMC6925144

[B14] Chikhi R. Sael L. Kihara D. (2011). Protein binding ligand prediction using moments-based methods” in protein function prediction for omics era. Dordrecht, Netherlands: Springer.

[B15] Christoffer C. Bharadwaj V. Luu R. Kihara D. (2021). LZerD Protein- Protein Docking Webserver Enhanced With de novo Structure Prediction. Front. Mol. Biosci. 8, 724947. 10.3389/fmolb.2021.724947 34466411 PMC8403062

[B16] Christoffer C. Chen S. Bharadwaj V. Adrinwale T. Kumar V. Hormati M. (2021). LZerD webserver for pairwise and multiple protein-protein docking. Nucleic Acids Res. 49, W359–W365. 10.1093/nar/gkab336 33963854 PMC8262708

[B17] Esquivel-Rodriguez J. Xiong Y. Han S. Guang S. Christoffer C. Kihara D. (2015). Navigating 3D electron microscopy maps with EM-SURFER. BMC Bioinforma. 16, 181. 10.1186/s12859-015-0580-6 PMC4448178 26025554

[B18] Eyrisch S. Helms V. (2007). Transient pockets on protein surfaces involved in protein-protein interaction. J. Med. Chem. 50, 3457–3464. 10.1021/jm070095g 17602601

[B19] Fukunishi Y. Mikami Y. Nakamura H. (2005). Similarities among receptor pockets and among compounds: Analysis and application to *in silico* ligand screening. J. Mol. Graph. Model. 24, 34–45. 10.1016/j.jmgm.2005.04.004 15950507

[B20] Gao M. Skolnick J. (2010). iAlign: a method for the structural comparison of protein-protein interfaces. Bioinformatics 25, 2259–2265. 10.1093/bioinformatics/btq404 PMC2935406 20624782

[B21] Gaulton A. Hersey A. Nowotka A. Bento A. P. Chambers J. Mendez D. (2017). The ChEMBL database in 2017. Nucleic Acids Res. 45, D945–D954. 10.1093/nar/gkw1074 27899562 PMC5210557

[B22] Glaser F. Pupko T. Paz I. Bell R. E. Bechor-Shental D. Martz E. (2003). ConSurf: Identification of functional regions in proteins by surface-mapping of phylogenetic information. Bioinformatics 19, 163–164. 10.1093/bioinformatics/19.1.163 12499312

[B23] Govindaraj R. G. Brylinski M. (2018). Comparative assessment of strategies to identify similar ligand-binding pockets in proteins. BMC Bioinforma. 19, 91. 10.1186/s12859-018-2109-2 PMC5845264 29523085

[B24] Halgren T. A. Murphy R. B. Friesner R. A. Beard H. S. Frye L. L. Pollard W. T. (2004). Glide: A new approach for rapid, accurate docking and scoring. 2. Enrichment factors in database screening. J. Med. Chem. 47, 1750–1759. 10.1021/jm030644s 15027866

[B25] Han X. Sit A. Christoffer C. Chen S. Kihara D. (2019). A global map of the protein shape universe. PLoS Comput. Biol. 15, 1006969. 10.1371/journal.pcbi.1006969 PMC6481876 30978181

[B26] Han X. Wei Q. Kihara D. (2017). Protein 3D structure and electron microscopy map retrieval using 3D-surfer2.0 and EM-SURFER. Curr. Protoc. Bioinforma. 60, 1–3. 10.1002/cpbi.37 PMC5726456 29220075

[B27] Heiden W. Moeckel G. Brickmann J. (1993). A new approach to analysis and display of local lipophilicity/hydrophilicity mapped on molecular surfaces. J. Comput.-Aided Mol. Des. 7, 503–514. 10.1007/BF00124359 8294943

[B28] Higurashi M. Ishida T. Kinosita K. (2009). PiSite: A database of protein interaction sites using multiple binding states in the PDB. Nucleic Acids Res. 37, D360–D364. 10.1093/nar/gkn659 18836195 PMC2686547

[B29] Hu B. Zhu X. Monroe L. Bures M. G. Kihara D. (2014). PL-PatchSurfer: A novel molecular local surface-based method for exploring protein-ligand interactions. Int. J. Mol. Sci. 15, 15122–15145. 10.3390/ijms150915122 25167137 PMC4200761

[B30] Ivanov A. A. Khuri F. R. Fu H. (2013). Targeting protein-protein interactions as an anticancer strategy. Trends Pharmacol. Sci. 34, 393–400. 10.1016/j.tips.2013.04.007 23725674 PMC3773978

[B31] Jin L. Wang W. Fang G. (2014). Targeting protein-protein interaction by small molecules. Annu. Rev. Pharmacol. Toxicol. 54, 435–456. 10.1146/annurev-pharmtox-011613-140028 24160698

[B32] Jurrus E. Engel D. Star K. Monson K. Brandi J. Felberg L. E. (2018). Improvements to the APBS biomolecular solvation software suite. Protein Sci. 27, 112–128. 10.1002/pro.3280 28836357 PMC5734301

[B33] Kihara D. Sael L. Chikhi R. Esquivel-Rodriguez J. (2011). Molecular surface representation using 3D Zernike descriptors for protein shape comparison and docking. Curr. Protein Pept. Sci. 12, 520–530. 10.2174/138920311796957612 21787306

[B34] Ko J. Park H. Seok C. (2012). GalaxyTBM: Template-based modeling by building a reliable core and refining unreliable local regions. BMC Bioinform 13, 198. 10.1186/1471-2105-13-198 PMC3462707 22883815

[B35] Kuhlman B. Dantas G. Ireton G. C. Varani G. Stoddard B. L. Baker D. (2003). Design of a novel globular protein fold with atomic-level accuracy. Science 302, 1364–1368. 10.1126/science.1089427 14631033

[B36] La D. Esquivel-Rodriguez J. Venkatraman V. Li B. Sael L. Ueng S. (2009). 3D-SURFER: Software for high-throughput protein surface comparison and analysis. Bioinformatics 25, 2843–2844. 10.1093/bioinformatics/btp542 19759195 PMC2912717

[B37] Li B. Turuvekere S. Agrawal M. La D. Ramani K. Kihara D. (2008). Characterization of local geometry of protein surfaces with the visibility criterion. Proteins 71, 670–683. 10.1002/prot.21732 17975834

[B38] Lipinski C. A. (2004). Lead- and drug-like compounds: The rule-of-five revolution. Drug Discov. Today Technol. 1, 337–341. 10.1016/j.ddtec.2004.11.007 24981612

[B39] Ma B. Elkayam T. Wolfson H. Nussinov R. (2003). Protein-protein interactions: Structurally conserved residues distinguish between binding sites and exposed protein surfaces. Proc. Natl. Acad. Sci. U. S. A. 100, 5772–5777. 10.1073/pnas.1030237100 12730379 PMC156276

[B40] Morelli X. Bourgeas R. Roche P. (2011). Chemical and structural lessons from recent successes in protein-protein interaction inhibition (2P2I). Curr. Opin. Chem. Biol. 15, 475–481. 10.1016/j.cbpa.2011.05.024 21684802

[B41] Needleman S. B. Wunsch C. D. (1970). A general method applicable to the search for similarities in the amino acid sequence of two proteins. J. Mol. Biol. 48, 443–453. 10.1016/0022-2836(70)90057-4 5420325

[B42] Pang B. Zhao N. Korkin D. Shyu C. R. (2012). Fast protein binding site comparisons using visual words representation. Bioinformatics 28, 1345–1352. 10.1093/bioinformatics/bts138 22492639

[B43] Park T. Baek M. Lee H. Seok C. (2019). GalaxyTongDock: Symmetric and asymmetric *ab initio* protein–protein docking web server with improved energy parameters. J. Comput. Chem. 40, 2413–2417. 10.1002/jcc.25874 31173387

[B44] Pearson W. R. (1991). Searching protein sequence libraries: Comparison of the sensitivity and selectivity of the smith-waterman and FASTA algorithms. Genomics 11, 635–650. 10.1016/0888-7543(91)90071-l 1774068

[B45] Rask-Andersen M. Almen M. S. Schioth H. B. (2011). Trends in the exploitation of novel drug targets. Nat. Rev. Drug Discov. 10, 579–590. 10.1038/nrd3478 21804595

[B46] Res I. Lichtarge O. (2005). Character and evolution of protein-protein interfaces. Phys. Biol. 2, S36–S43. 10.1088/1478-3975/2/2/S04 16204847

[B47] Sael L. Kihara D. (2012). Detecting local ligand-binding site similarity in nonhomologous proteins by surface patch comparison. Proteins 80, 1177–1195. 10.1002/prot.24018 22275074 PMC3294165

[B48] Sael L. Kihara D. (2010). Improved protein surface comparison and application to low-resolution protein structure data. BMC Bioinforma. 11, S2. 10.1186/1471-2105-11-S11-S2 PMC3024873 21172052

[B49] Sael L. Kihara D. (2010). Binding ligand prediction for proteins using partial matching of local surface patches. Int. J. Mol. Sci. 11, 5009–5026. 10.3390/ijms11125009 21614188 PMC3100846

[B50] Scannell J. W. Blanckley A. Boldon H. Warrington B. (2012). Diagnosing the decline in pharmaceutical R&D efficiency. Nat. Rev. Drug Discov. 11, 191–200. 10.1038/nrd3681 22378269

[B51] Shin W. H. Christoffer C. W. Kihara D. (2017). *In silico* structure-based approaches to discover protein-protein interaction-targeting drugs. Methods 131, 22–32. 10.1016/j.ymeth.2017.08.006 28802714 PMC5683929

[B52] Shin W. H. Christoffer C. W. Wang J. Kihara D. (2016). PL-PatchSurfer2: Improved local surface matching-based virtual screening method that is tolerant to target and ligand structure variation. J. Chem. Inf. Model. 56, 1676–1691. 10.1021/acs.jcim.6b00163 27500657 PMC5037053

[B53] Shin W. H. Kihara D. (2019). Predicting binding poses and affinity ranking in D3R Grand Challenge using PL-PatchSurfer2.0. J. Comput.-Aided Mol. Des. 33, 1083–1094. 10.1007/s10822-019-00222-y 31506789

[B54] Shin W. H. Kihara D. (2018). Virtual ligand screening using PL-PatchSurfer2, a molecular surface-based protein-ligand docking method. Methods Mol. Biol. 1762, 105–121. 10.1007/978-1-4939-7756-7_7 29594770

[B55] Shin W. H. Kumazawa K. Imai K. Hirokawa T. Kihara D. (2020). Current challenges and opportunities in designing protein–protein interaction targeted drugs. Adv. Appl. Bioinform. Chem. 13, 11–25. 10.2147/AABC.S235542 33209039 PMC7669531

[B56] Shin W. H. Zhu X. Bures M. G. Kihara D. (2015). Three-dimensional compound comparison methods and their application in drug discovery. Molecules 20, 12841–12862. 10.3390/molecules200712841 26193243 PMC5005041

[B57] Shulman-Peleg A. Shatsky M. Nussinov R. Wolfson H. J. (2007). Spatial chemical conservation of hot spot interactions in protein-protein complexes. BMC Biol. 5, 43. 10.1186/1741-7007-5-43 17925020 PMC2231411

[B58] Stumpf M. P. H. Thorne T. de Silva E. Stewart R. An H. J. Lappe M. (2008). Estimating the size of the human interactome. Proc. Natl. Acad. Sci. U.S.A. 105, 6959–6964. 10.1073/pnas.0708078105 18474861 PMC2383957

[B59] Toogood P. L. (2002). Inhibition of protein-protein association by small molecules: Approaches and progress. J. Med. Chem. 45, 1543–1558. 10.1021/jm010468s 11931608

[B60] Vassilev L. T. Vu B. T. Graves B. Carvajal D. Podlaski F. Filipovic Z. (2004). *In vivo* activation of the p53 pathway by small-molecule antagonists of MDM2. Science 303, 844–848. 10.1126/science.1092472 14704432

[B61] Venkatraman V. Chakravarthy P. R. Kihara D. (2009). Application of 3D Zernike descriptors to shape-based ligand similarity searching. J. Cheminform. 1, 19. 10.1186/1758-2946-1-19 20150998 PMC2820497

[B62] Venkatraman V. Sael L. Kihara D. (2009). Potential for protein surface shape analysis using spherical harmonics and 3D Zernike descriptors. Cell Biochem. Biophys. 54, 23–32. 10.1007/s12013-009-9051-x 19521674

[B63] Xiong Y. Esquivel-Rodriguez J. Sael L. Kihara D. (2014). 3D-SURFER 2.0: Web platform for real-time search and characterization of protein surfaces. Methods Mol. Biol. 1137, 105–117. 10.1007/978-1-4939-0366-5_8 24573477

[B64] Zhang Y. Skolnick J. (2004). Scoring function for automated assessment of protein structure template quality. Proteins 52, 702–710. 10.1002/prot.20264 15476259

[B65] Zhu H. Sommer I. Lengauer T. Domingues F. S. (2008). Alignment of non-covalent interactions at protein-protein interfaces. PLoS One 3, e1926. 10.1371/journal.pone.0001926 18382693 PMC2274958

[B66] Zhu X. Shin W. H. Kim H. Kihara D. (2016). Combined approach of patch-surfer and PL-PatchSurfer for protein-ligand binding prediction in CSAR 2013 and 2014. J. Chem. Inf. Model. 56, 1088–1099. 10.1021/acs.jcim.5b00625 26691286 PMC5005040

[B67] Zhu X. Xiong Y. Kihara D. (2015). Large-scale binding ligand prediction by improved patch-based method Patch-Surfer2.0. Bioinformatics 31, 707–713. 10.1093/bioinformatics/btu724 25359888 PMC4341070

